# Exploring the benefits of online teaching and learning within a nursing programme at a higher education institution in Gauteng

**DOI:** 10.4102/curationis.v49i1.2812

**Published:** 2026-06-29

**Authors:** Sanele E. Nene, Agnes Makhene

**Affiliations:** 1 Department of Nursing, Faculty of Health Sciences, University of Johannesburg, Johannesburg, South Africa

**Keywords:** benefits, online, teaching and learning, higher education, institution, nursing education

## Abstract

**Background:**

Globally, there is an abrupt shift from in-person to online teaching and learning, and institutions of higher education should ensure that no students are left behind from this revolution. A total of 73% of nurses in South Africa are Baby Boomers and Generation X, meaning they were born before technology and are struggling to navigate online teaching and learning platforms made accessible by the institution.

**Objectives:**

This study aimed to explore and describe the lived experiences of post-basic student nurses on the benefits of online teaching and learning at a higher education institution.

**Method:**

A qualitative, exploratory, descriptive, contextual research design and phenomenological approach were adopted in this study. Data were collected from 10 participants using individual interviews and analysed following thematic analysis.

**Results:**

One central theme and three main themes emanated from this study. A central theme was that online teaching and learning offers efficient and flexible education for nursing students, while improving their technological competencies. Main themes are that online teaching and learning: (1) is convenient for students, (2) saves time and is cost effective and efficient for students, and (3) is flexible and improves students’ knowledge and skills in technology.

**Conclusion:**

The institutions of higher education should realise the benefits of online teaching and learning to improve the quality of nursing graduates.

**Contribution:**

This article clarifies the benefits of online teaching and learning for nursing education in a South African context.

## Introduction

It is clear from the developments that are happening in teaching and learning that the higher education institutions in the developing countries, such as South Africa, are joining the first-world countries to embrace the fourth industrial revolution (Ally & Wark 2020). This is observed through the abrupt shift from traditional to online teaching and learning, ensuring that educational activities are relevant for the current generation and sustained for the next generation (Suliman et al. 2021). Globally, online teaching and learning is a critical historical epoch, and it has revealed a serious digital divide amid the fourth industrial revolution buzz and a looming fifth industrial revolution (Moyo 2023).

The teaching and learning institutions that were already advanced in online teaching and learning and agile enough to swiftly move to this platform performed better than those who were still behind on this notion during the coronavirus disease 2019 (COVID-19) pandemic (Haldar et al. 2020). According to Suliman et al. (2021), the pandemic frustrated the educators and students, especially adult students who were born before technology was used on a large scale in the market, as teaching and learning had to be adapted to accommodate the circumstances of the pandemic and to ensure its continuity. The Organisation for Economic Co-operation and Development (2020) posited that online teaching and learning is not only conducted remotely but can also be used to complement the traditional pedagogies in the physical classroom, and this is referred to as blended learning. Xu, David and Kim (2018) and Nene and Hewitt (2023) noted that South African higher education institutions need to evolve with the changing times to improve the quality of teaching and learning; consequently, embracing the online teaching and learning benefits is important for these institutions.

In South Africa, the nursing schools within the higher education institutions are tasked by the Council of Higher Education and the South African Nursing Council to develop world-class nurses who will be responsive to the healthcare demands of this time (Muller, Bezuidenhout & Jooste 2021). They are expected to embrace the benefits of online teaching and learning, which include an increase in access to education, flexibility, minimising costs, increased collaborations, and many more (Moyo 2023; Nene & Hewitt 2023). Most nurses are senior citizens and are challenged by technology; hence, only in 2023 that the curriculum for nurses included technology-related exit level outcomes (Department of Health, Regulation Number 635 of 2020). Considering the benefits of online learning, which ensure that a global nurse is developed, the South African higher education institutions should prioritise and fast-track online teaching and learning and closely monitor its effectiveness (Mahyoob 2020).

### Problem statement

During the COVID-19 pandemic, the higher education institutions had to adapt to online learning. Over a period of 24 months, during and post the pandemic, the researcher has observed that in an institution where he was studying, most post-basic student nurses and educators were struggling with technology; they were unable to navigate online platforms such as the learner management system, Moodley, and Blackboard made accessible by the institution. This observation was evidenced by expressions from most students reporting that they are not performing well because they are slow to use technology or struggling to use the online learning platform. Other lecturers during online class sessions would inform students that they are struggling to navigate online teaching and learning platforms. The student nurses are self-funders and have challenges with connection to online learning because of the related costs such as internet connection, laptops and other infrastructure. Marinoni, Van’t Land and Jensen (2020) elicited that online teaching and learning is overwhelming for nursing students and educators, as computer literacy was not inclusive in the previous nursing curriculum since the advent of the fourth industrial revolution. Coman et al. (2020) revealed that most nurse educators are resistant to change, and this poses a difficulty in transforming the perceptions of students about online teaching and learning. There is clearly an inequality in access to technology infrastructure in South Africa, which hinders nursing students from exploiting the benefits of online teaching and learning. These include limited technology support, inadequate coordination, general shortage of computers, difficulties related to internet access, data reliability and affordability (Agu et al. 2021; Mahyoob 2020).

Online teaching and learning are conspicuously an additional workload for nurse educators as they are required to re-package learning content to suit online modes. Isabirye and Dlodlo (2014) and Heng and Sol (2020) affirmed that in addition to the inequality concerns, there is a lack of preparation and administration of online teaching and learning in higher education institutions. In their study, Abbasi et al. (2020) concluded that some student nurses preferred contact sessions over online learning, and 34% opted to forfeit exams after totally taking online classes because they felt unprepared by online teaching and learning sessions. The following research question emanated from the problem statement. *What are post-basic student nurses’ lived experiences on the benefits of online teaching and learning within a higher education institution in Gauteng?*

### Aim and objectives

The aim of the study was to understand the lived experiences of post-basic student nurses on the benefits of online teaching and learning at a higher education institution.

The objectives of the study were to explore and describe the lived experiences of post-basic student nurses on the benefits of online teaching and learning at a higher education institution, and to embrace these benefits to improve the quality of the nursing education system.

### Setting

This study was conducted in a specific higher education institution in Gauteng, offering nursing undergraduate and postgraduate qualifications through a hybrid teaching and learning approach to facilitate teaching and learning.

## Research methods and design

A qualitative, exploratory, descriptive, contextual research design and phenomenological approach were adopted in this study to answer the research question. This design and approach were deemed relevant as they focus on understanding the phenomenon of interest through the exploration of reality and naturalistic observations of the participants. The following research methodologies were used in this study.

### Population and sampling

The population of the study was nursing students within a specific higher education institution, using an online teaching and learning platform, and the sample was the post-basic students enrolled for the qualification Baccalaureus Curationis (Educationis Et Administrationis) Degree in Occupational Health Nursing. This study was conducted using the last cohort of students of this qualification, as it has been phased out because of curriculum reform. Nine females and one male student aged between 32 and 51 participated in this study and were selected using a non-probability purposive sampling method to ensure that they are relevant for the study.

### Data collection

Data were collected by the researcher using face-to-face, unstructured or virtual individual interviews between 01 October 2022 and 21 December 2022, and participants were given a choice to choose a method of interview that was suitable or convenient for them. Face-to-face interviews were conducted in a coffee shop convenient for the participant, while virtual interviews were carried out at the comfort of the participants’ homes. An information letter was shared with the participants using an email, and questions were addressed, interested participants completed informed consent. Emails of the participants were requested using a WhatsApp study group, which a researcher was part of as they were in the same class. The participants who were willing to participate in the study were requested to respond with their emails directly to the researcher in a private conversation between the researcher and the prospective participant. Participants were allowed to choose a data collection method that was suitable for them, and those who chose virtual interviews mentioned that they had uncapped Wi-Fi and were comfortable with the costs of online interviews. The date, location, and timing of each interview were coordinated in accordance with participants’ availability, with each session having a duration of approximately 45 min to 60 min. English was used for the interviews as all the participants were proficient in the use of the English language, and none of them had a challenge communicating in English. Field notes were recorded during the interviews, and data saturation was reached by the tenth participant. To confirm data saturation, an additional interview was conducted, which yielded no new information. The researcher set aside preconceived assumptions and personal biases by using a reflective journal to document their assumptions before and during the study, preventing them from influencing data interpretation.

### Data analysis

Data were analysed using Giorgi’s descriptive phenomenological thematic data analysis method (Giorgi, Giorgi & Morley 2017). The researcher extracted the key themes and patterns from the experiences of the participants. An independent coder with vast expertise in qualitative research and nursing education was appointed to confirm the findings that emerged, and a consensus meeting on the findings was held.

### Trustworthiness

The following measures to ensure trustworthiness of the study as suggested by Lincoln et al. (2018) were followed. Credibility was ensured by collecting data using face-to-face and virtual individual interviews and recording field notes during the interviews. All interviews were audio-recorded and transcribed verbatim to maintain the authenticity of the findings. The independent coder confirmed the results, and regular supervision sessions were held by the supervisors of the researcher and the researcher to ensure confirmability and dependability of the findings. Dense information was discussed on the population of the study, the context, research design and methods adopted to conduct the study, to allow other researchers to transfer the findings of the study to other similar settings, should they anticipate doing that.

### Ethical considerations

The following ethical principles were upheld in this study: permission to conduct the study, autonomy, privacy, confidentiality, risks and benefits (Gray & Grove 2021). The permission to conduct the study was sought and granted by the University of Johannesburg Faculty of Health Sciences Research Ethics Committee (REC-1590-2022), where the study was conducted. The participants were approached at their own liberty, and they voluntarily granted permission to participate in the study and that of audio-recording in writing. In addition, participants were informed that they can withdraw from participating any time and no penalties will be imposed on them for doing so. Privacy and confidentiality were ensured by assigning code names to replace the identities of the participants in the transcription, while the raw data of the study were safely kept in a locked and security-protected folder. The raw data will be safely discarded after 5 years of publication of the results of this study. There were no risks envisaged for participating in this study; however, participants were informed that should they experience any anxiety or emotional triggers because of the study, they should report it, and the researcher would refer them to the psychologist of the Higher Education Institution where the study was conducted. The findings of the study were shared with the participants and management of this institution for implementation.

## Results

One central theme and three main themes emanated in this study and are presented in Table 1.

**TABLE 1:** Central theme and main themes.

Variable	Description
Central theme	A central theme was online teaching and learning offer efficient and flexible education for nursing students, while improving their technological competencies.
Theme 1	Online teaching and learning is convenient for students.
Theme 2	Online teaching and learning saves time and is cost effective and efficient for students.
Theme 3	Online teaching and learning is flexible and improves students’ knowledge and skills in technology.

### Online teaching and learning provide flexible, efficient education while enhancing technological competencies

The participants mentioned that online teaching and learning provided them with efficient and flexible education while improving their technological abilities. This is confirmed by the following quotations from the participants:

‘I enjoyed online learning I must say, accessing the recordings and then taking notes down. Unlike when you are in class, if you have missed it, you have missed it. So, with the recordings, you can always go back and listen repeatedly.’ (P5, 30 years, female)

Another participant, taking a deep breath and looking emotional, said:

‘Hmm, yes, I think we learned a lot about this online thing. We became experts with typing and knowing how to schedule Zoom meetings. We ended up knowing how to record things and how to look for recordings.’ (P3, 50 years, female)

#### Theme 1: Online teaching and learning is convenient for students

In the first main theme of the study, the participants reported that online teaching and learning was convenient for them, and they are enjoying this educational transformation as it enables them to keep and access their records anytime. Other participants added that they did not have to drive to campus to access class, but they managed to attend class at the comfort of their home:

‘So, for me, it was very convenient, I feel like it was helping me. I was doing well with online learning, and I enjoyed it. I did not have any major challenges with it.’ (P5, 30 years, female)

‘The nice thing also about online teaching and learning is that you could keep records, and you can go over and over again ... So you know you have a recording, you can listen to it while you are driving, while you are cooking.’ (P7, 35 years, female)

‘I fell in love with it because, remember, I was staying far from the university. I was driving every time to attend class now I was doing classes at home ... So online teaching and learning helped me a lot because now I was waking up at normal times, I was relaxed, had my breakfast knowing I would have my classes in the house [*smiling*].’ (P3, 50 years, female)

‘So, I was happy that I was not supposed to drive long distances anymore [*looking excited*].’ (P4, 39 years, female)

#### Theme 2: Online teaching and learning saves time and is cost effective and efficient for students

The participants mentioned that learning saves time and reduces costs as they do not have to travel to university or to spend money to travel to attend class:

‘I liked that I did not have to spend money to attend varsity. I can attend classes even at work.’ (P6, 42 years, female)

‘It is nice. It is timesaving, it saves money, you do not spend, and you do not go to school or sit in the classroom. You are at home [*smiling and using both hands to explain*].’ (P2, 51 years, female)

One participant stated:

‘It appeared OK because, with travel costs, you know, I saved money. I saved much time and the effort of going to school.’ (P7, 35 years, female)

Another summed it up by stating:

‘I liked that I did not have to spend money to go to varsity. It really saved me time.’ (P4, 39 years, female)

#### Theme 3: Online teaching and learning is flexible and improves students’ knowledge and skills in technology

The participants also expressed that online teaching and learning is flexible, allowing them to attend class while at work or busy with their daily activities at home. On the other hand, the participants alluded that this teaching and learning method improved their technological competencies as they are now able to do video calls and use their computers better such as typing faster:

‘… so, I’ll be able to attend classes even when I am at work. I also asked my manager to say that I want to go and attend class online, and sometimes, even if I had something to attend to at home, I would be able to attend class while doing those things at home.’ (P6, 42 years, female)

‘You see, after teaching and online learning, I think it encouraged me because when I do a WhatsApp call, I do video calling now. It has taught me if you want to talk with somebody, there is a way of talking to them, especially if you want to see them face to face.’ (P2, 51 years, female)

‘Hmm, ok, most of us are not computer literate. So online learning challenges us to learn more about computer things so that we are fast when typing those things [*looking concerned and placing the right hand on the cheek*].’ (P6, 42 years, female)

## Discussion

Online teaching and learning provided nursing students with an efficient and flexible mode of education while simultaneously enhancing their technological competencies. Malematja, Nkosi and Nene (2025) and Makhene, Nene and Mhlongo (2025) argue that higher education in South Africa is undergoing significant transformation across faculties and disciplines through the prioritisation of online teaching and learning. Similarly, South African Nursing Council (2021) asserts that the nursing profession must evolve by developing digitally competent nurses capable of responding effectively to healthcare challenges in the digital era.

The findings of this study indicate that online teaching and learning were perceived as both convenient and engaging by student nurses. Supporting this observation, Wu et al. (2021) reported that contemporary students increasingly embrace online teaching and learning, largely because they perceive it as more convenient than traditional classroom-based environments. Furthermore, Osika et al. (2022) emphasise that teaching and learning approaches should prioritise student convenience to facilitate the achievement of desired learning outcomes. Consistent with the view that online learning offers significant convenience to students, Du Plessis and Van der Westhuizen (2021) and Rubeena (2021) concluded that the convenience associated with online learning contributes positively to students’ academic performance.

In their study on online teaching and learning, Ranganathan et al. (2021) and Yazdannik, Mohamadirizi and Nasr-Esfahani (2020) reported that 67.57% of students were extremely satisfied with online learning. However, Rohde et al. (2022) argue that it is misleading to assume that online teaching and learning are without challenges. Despite these challenges, students still demonstrate satisfaction with the opportunity to experience this significant transition in their learning environments. Furthermore, O’Doherty et al. (2018) identified several barriers associated with online teaching and learning, including reliability and connectivity issues, limited technical skills, time constraints, inadequate infrastructure, the absence of clear institutional strategies and support, as well as negative attitudes among stakeholders. Despite these challenges and barriers, the student nurses in this study were still able to recognise the potential and benefits of online teaching and learning. Nevertheless, it is essential for higher education institutions and nurse educators to develop and implement effective strategies to mitigate these challenges and barriers to ensure the delivery of effective teaching and learning.

The students also reported that online teaching is cost effective and efficient for them. They also mentioned that they were not required to travel to university for classes. Oxford School of Business (2023), Chandrasiri and Weerakoon (2021), Dinh and Nguyen (2022) affirm that students receive cost-saving benefits from online learning, such as reduced tuition fees, absence of relocation and travelling costs, flexible scheduling, access to free or low-cost educational resources, and reduced networking expenses. Online teaching and learning experiences of students revealed that more than 65% of the students in a specific higher education institution were excited by this method that it saves time and allows them to perform other tasks in between their classes (Subedi et al. 2020). The cost of living is increasing, so it is appreciated that now students can embrace the benefits of technologies such as online teaching and learning, which include learning without incurring travel expenses (Nene & Hewitt 2023). Bates (2019) further stated that online teaching and learning could and should drastically reduce the cost of higher education, to enable more accessibility to the students from marginalised communities.

The students found online teaching and learning flexible and improved their knowledge and skills in technology. In a Theory of Connectivism Learning, Siemens (2005) argues that online teaching and learning is most beneficial to the digital age students as it enables them to access the learning content wherever they are, while improving their technology skills. Nene and Hewitt (2023) reveal that based on the previous curriculum of South African student nurses, which did not consist of technology skills, current nurses lack understanding and application of technology in their practice, and this should be addressed promptly. Recently, the South African Nursing Council and the Council of Higher Education reached a consensus to review the South African nursing curriculum (SANC 2021). In this review, the technological skills for nurses are incorporated; consequently, at the end of the qualification, nurses are expected to demonstrate awareness of technology and techniques used to connect with remote external environments. This technological skill is essential to develop technologically savvy, future-ready nurses who can respond effectively to healthcare challenges of this digital age (Bates 2019).

In support of the findings of this study, Enakrire (2024) states that technological skills such as computer literacy are now an essential commodity required by every individual globally and most importantly educators because of the dynamics involved in working in virtual spaces, where many online educational resources are used to support online teaching tactics, which are explicit to students and other colleagues. The study on online teaching and learning at a South African university by Maphalala and Adigun (2021) affirm this by concluding that technical support and training for online learning, information communication technology infrastructure, internet accessibility, and uptake of online learning ensure the realisation of flexible online learning. Enakrire (2024) argues that technological skills have become fundamental in nursing education because of the increasing use of new technological tools of web-based management systems, laptops, digital tablets, learning management systems, webinars, and the applications of Zoom, Microsoft Teams, and WhatsApp manufactured daily. Ngubane-Mokiwa and Letseka (2015) and Ngubane-Mokiwa (2017) posit that these skills revolutionise nurse educators to access and make optimal use of online educational resources, thereby facilitating activities of teaching and learning and research practices to students irrespective of their geographical location.

The findings of this study reveal that online teaching and learning improves students’ knowledge and skills in technology, making online learning more flexible. However, there are various technologies used in hospitals that require nurses to be trained to have such knowledge and skills, and online teaching and learning can be the starting point to sharpen these skills (Nene & Hewitt 2023). Equipping nursing students with technological knowledge and skills does not mean that online teaching and learning methods will replace the physical ones but will instead augment them for greater efficiency (Enakrire 2024).

### Limitations

The findings of this study are not generalisable as they are limited to the sample of nursing students enrolled for the qualification Baccalaureus Curationis (Educationis Et Administrationis) Degree in Occupational Health Nursing in South Africa. This, however, offers insights for future research on the same topic being expanded to other contexts in the developing and developed countries. This study was conducted during peak times of the COVID-19 pandemic; hence, it was difficult to access as many participants as possible.

### Recommendations

The recommendations for policy, practice and nursing education that emanated from this study are presented below. A policy and guidelines on online teaching and learning for nursing education institutions should be developed to ensure a seamless facilitation of online teaching and learning in nursing. The nursing education institutions should devise an orientation programme for online teaching and learning for students and lecturers. Higher education institutions should work with stakeholders, such as the government and network providers, to allocate the resources required for online teaching and learning. The nursing education institutions should ensure that lecturers are trained to effectively use the evolving online teaching and learning platforms. Digital skills education should be incorporated into the curriculum of nursing students.

## Conclusion

South Africa is now in the digital age, and nursing education is also not left behind in this current revolution. This is evidenced by the benefits of online teaching and learning that emerged in this study. The institutions of higher education should embrace online teaching and learning on a larger scale while providing adequate support to nursing students to improve the quality of nursing graduates and to ensure that these graduates are not technologically excluded and are at a world-class level. It can be considered that the future of nursing education is digital; therefore, the right infrastructure and policies should be put in place to be better prepared for the future and to ensure seamless transition (Maphalala & Adigun 2021).
